# Improved executive function and sleep quality in preteens with high-functioning autism following a structured physical activity program

**DOI:** 10.3389/fpsyt.2025.1726809

**Published:** 2026-03-16

**Authors:** Miriam Richter, Marie K. Taylor, Sofia Åkerlund, Sofia Backman, Sofia Krili, Björn Axel Johansson, Olof Rask, Christine T. Ekdahl

**Affiliations:** 1 Division of Clinical Neurophysiology, Department of Clinical Sciences, Lund University, Lund, Sweden; 2 Division of Child and Adolescent Psychiatry, Department of Clinical Sciences, Lund University, Lund, Sweden; 3 Clinical Neurophysiology, Department of Medical Imaging and Physiology, Skåne University Hospital, Lund, Sweden; 4 Epilepsy Center, Department of Clinical Sciences, Lund University, Lund, Sweden

**Keywords:** actigraphy, ASD, autism spectrum disorder, CANTAB, cognition, physical activity, sleep

## Abstract

**Purpose:**

Children with autism spectrum disorder (ASD) frequently exhibit atypical cognitive profiles, sensory processing challenges, sleep disturbances, and co-occurring psychiatric and somatic conditions. This exploratory prospective study, with a single-arm pre-post design, investigated the effects of a 10-12 weeks structured physical activity program on psychological symptoms, cognitive function, and sleep in children aged 10–14 years with high-functioning ASD.

**Methods:**

Pre- and post-intervention assessments included parent-reported questionnaires, computerized cognitive testing using Cambridge Neuropsychological Test Automated Battery (CANTAB), and actigraphy-based sleep-wake monitoring.

**Results:**

Sixteen children completed the intervention (n = 12 boys; median age 11.5 years). Following the intervention, parents reported improvements in executive functioning as measured by the Behavior Rating Inventory of Executive Function, and in sensory processing as assessed by the Child Sensory Profile. Objective cognitive testing indicated tentative improvements in executive functioning, particularly in planning and problem-solving, on the Stockings of Cambridge (SOC) task in CANTAB. Actigraphy data indicated improved sleep quality, reflected by a reduced fragmentation index.

**Conclusion:**

A structured physical activity program may improve executive functioning (as perceived by parents and supported by objective testing), enhance sensory processing, and improve sleep quality in children with high-functioning ASD who are not regularly physically active.

## Introduction

Autism Spectrum Disorder (ASD) is a developmental neuropsychiatric condition characterized by persistent deficits in social communication and interaction, alongside restricted and repetitive patterns of behavior, interests, or activities (1). Approximately 1.5% of the population is affected by ASD (2–4) and psychiatric comorbidities are frequent, including other neuropsychiatric and affective disorders (5, 6), as well as somatic comorbidities such as epilepsy (7, 8) and obesity (9).

Individuals with ASD often exhibit an atypical cognitive profile, with impairments in social cognition, sensory processing, perception, attention shifting, information processing, and executive function, i.e. planning, mental flexibility, working memory, and impulse control (10–14). Children and adolescents with ASD are also reported to experience sleep disturbances at significantly higher rates than the general population (15, 16). For example, ASD has been linked to bedtime resistance and night awakenings, which contribute to difficulties in falling asleep and returning to sleep (17).

Previous studies have shown that physical activity benefits several domains of cognition in children, including working memory, attention and flexibility (18, 19) as well as improving sleep quality (20–22). However, children with ASD are often more sedentary and less physically active than their neurotypical peers (23–26). Evidence suggests that promoting physical activity among children and adolescents with ASD can improve physical health, enhance motor skills, and support the development of social and communicative abilities (27, 28). Physical activity has also been associated with improvements in school performance, stereotypic behaviors, cognition (29–32), and sleep quality (33).

Randomized controlled trials of physical activity interventions in children with ASD have demonstrated improvements in parent-reported psychological outcomes, including improved emotional regulation and reduced behavioral problems after a jogging program measured by the Emotion Regulation Checklist and the Child Behavior Checklist (34), improved social responsiveness after a sensory integration–based sports program measured by Social Responsiveness Scale (SRS) (35), and improved executive functioning after physical training measured by the Behavior Rating Inventory of Executive Function (BRIEF) (36).

However, although the number of studies in this area is increasing, they consistently highlight the need for further research (37), particularly studies with stratified and objectively confirmed ASD diagnoses (29) and clearly defined intervention parameters to enable robust assessment of associated psychological symptoms, cognitive outcomes, sleep, and circadian rhythms.

In this exploratory prospective study, with a single arm pre-test/post-test design, we examined whether a 10–12 weeks physical pulse-raising (aerobic) activity program could affect psychological symptoms, cognitive functions, and sleep in children with high-functioning ASD.

## Materials and methods

### Project outline and participants

**Figure 1:**
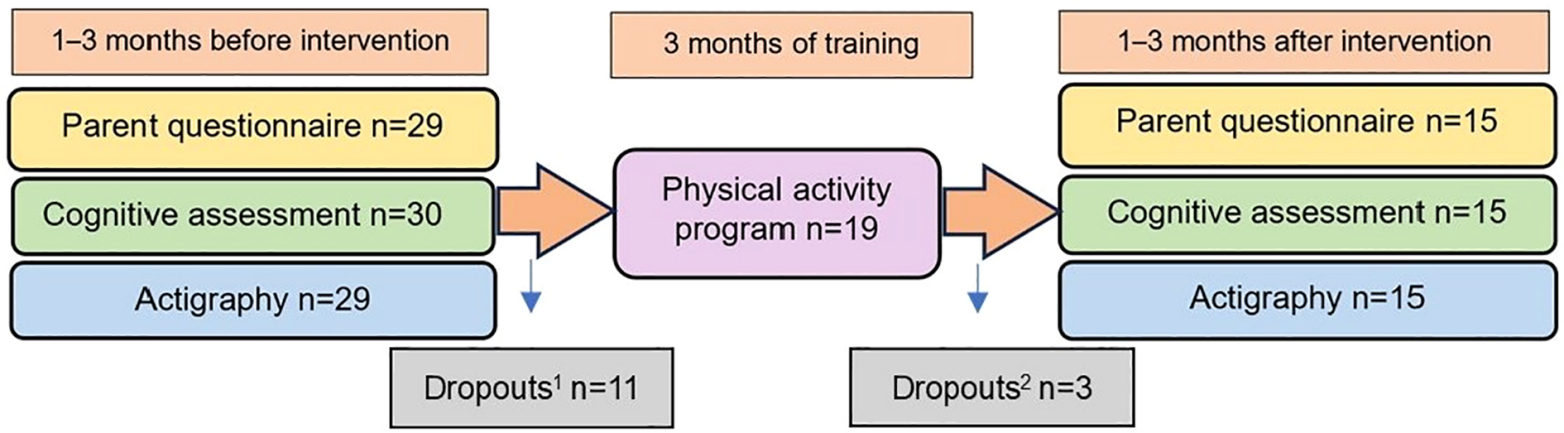
Project outline. The project outline presents the study timeline, detailing its three main phases: pre-intervention assessments, the physical activity intervention period, and post-intervention assessments. It also indicates the number of participants (n) involved in each phase. Dropouts^1^ (n = 11) refers to children who declined to participate in the physical activity program after the initial assessments. Dropouts^2^ (n = 3) refers to children who attended only one session of the physical activity program before withdrawing and were therefore not included in the post-intervention assessments. In addition, parent questionnaire and actigraphy data were unavailable pre-intervention for n=1 participant, and data from n=1 participant was missing for each of three post-intervention assessments (three different patients).

This project was an exploratory prospective single arm study that included pre-intervention assessments, 10–12 weeks of physical activity and post-intervention assessments (**Figure 1**). The assessments consisted of parent questionnaires, cognitive assessments and actigraphy recordings. Recruitment was conducted over the period 2020-2022. Participants were identified through patient medical records and their contacts at the Child and Adolescent Psychiatric Clinic or the Child and Adolescent Habilitation Clinic, Region Skåne, Sweden. Children with a clinically established ASD diagnosis documented in their medical records were recruited from Lund, Malmö and surrounding areas in southern Sweden. ASD severity was documented in the medical chart alongside the diagnosis, according to DSM-5 criteria: Level 1= requiring support; Level 2= requiring substantial support; Level 3= requiring very substantial support. The authors contacted parents directly by phone to invite families to take part in the study. Children with high-functioning ASD were primarily recruited, operationalized as individuals with ASD level 1–2 according to DSM-5 and exhibiting average cognitive ability and spoken language. Inclusion criteria comprised a confirmed ASD diagnosis at level 1-2, age between 10–14 years, and the ability to understand and communicate using spoken language. Exclusion criteria were diagnosis of moderate-severe intellectual disability, traumatic brain injury within the last 6 months, active systemic autoimmune disease or neurodegenerative disorders. Demographic data and medical history, including diagnoses, radiological findings and perinatal data, were obtained from medical records. Child-adjusted information on the assessments in the study was provided prior to inclusion. Written informed consent was obtained from the legal guardians. The children were simultaneously enrolled in an additional exploratory cross-sectional study pre-intervention, in which they underwent blood sampling for measurements of immune factors in serum (*manuscript under review*).

### Parent questionnaires

The children’s parents completed a series of clinical assessment questionnaires to evaluate psychiatric symptoms and psychological well-being in their children. The questionnaires administered included the Autism Spectrum Screening Questionnaire (ASSQ), whose psychometric properties have been examined in several studies, demonstrating good test–retest reliability for parent ratings (0.96) as well as a sensitivity (0.91) and a specificity (0.86) in a Norwegian study of school-aged children (7–9 years), using a cutoff score of 17 (38–41). Of note, the ASSQ was not used to determine whether participants met criteria for an autism diagnosis, but to assess the presence and severity of parent-reported ASD-related symptoms. The BRIEF, Second Edition (BRIEF-2), has been psychometrically evaluated in children and adolescents with and without Attention-Deficit/Hyperactivity Disorder (ADHD) and ASD (42, 43). The Child Sensory Profile, Second Edition (CSP-2), has demonstrated acceptable psychometric properties in typically developing children as well as in children with ASD (14, 44–46). SRS, Second Edition, has been validated in both typically developing children and children with ASD (47). The Strength and Difficulties Questionnaire (SDQ) has been psychometrically evaluated in large community samples of children aged 3–17 years (48, 49). Finally, the Swanson, Nolan, and Pelham 26-item Rating Scale (SNAP-IV) has been evaluated in children with and without ADHD (50, 51). All questionnaires are standardized and widely used in child and adolescent psychiatric clinical settings. For further description of each questionnaire, see **Supplementary Table 1**. In the BRIEF and SRS questionnaires, individual item scores could exceed 90. When this occurred, the values were truncated to a predefined maximum of 90 to facilitate statistical analyses.

### Cognitive assessment

For cognitive assessment, all children underwent a selection of tests from the computerized Cambridge Neuropsychological Test Automated Battery (CANTAB), developed at the University of Cambridge, and marketed by Cambridge Cognition Ltd. The CANTAB battery includes multiple tests targeting different aspects of cognitive function. All tests are described and shown in videos at the website (https://cambridgecognition.com). The assessment was facilitated by a psychologist experienced with both the test and children with ASD. Five distinct CANTAB subtests were conducted to assess various cognitive domains that might be impacted in individuals with ASD: intra-extra dimensional set shift (IED), paired association learning (PAL), reaction time (RTI), stockings of Cambridge (SOC) and spatial working memory (SWM). IED tests mental flexibility, PAL tests visual memory and new learning strategies, RTI tests reaction time and speed, SOC tests planning and SWM working memory. For further description of each test, see **Supplementary Table 2**. CANTAB batteries have been used and validated in previous studies for assessing neuropsychological function in children (52, 53), including children with ASD (54). The tests are sensitive to cognitive dysfunction across the spread of ASD severities and across age ranges (54–57).

### Actigraphy

Sleep–wake patterns were evaluated using actigraphy. The children were asked to wear an actigraphy watch (MotionWatch 8, CamNtech, UK), which monitors movement and light exposure, for 5–7 days. Minimum of three weekdays were accepted for inclusion in the analysis to minimize variability related to differences in sleep schedules between schooldays and weekends, ensuring more representative estimates of typical sleep patterns. This duration is considered the minimum required to obtain acceptable reliability for sleep measurements in children (58). For valid sleep measurement, only nights with continuous actigraphy recording across the entire night were included. Daytime data were summarized as available hours of recorded activity. Information about sleep and activity habits as well as ongoing pharmacological treatments were obtained from a parent questionnaire before the first actigraphy assessment. Actigraphy data was analyzed by two senior consultants in clinical neurophysiology, with the CamNtech Motionware 1.2.5 software. Participants were told to press the bottom on the clock when they turned the light off in the evening, and when they left the bed in the morning. During these days, the parents were also asked to write down at what time their children went to bed, how long after they fell asleep, when they woke up and when they got up. The notes, together with the actigraphy data, were then weighed together by the authors to estimate the following parameters for each child: median lights out, sleep latency, assumed sleep, fragmentation index, wake bouts, and total activity score during daytime. Sleep latency was measured as the duration between the time point the child tries to go to sleep (lights out) and the start of the first block of immobility (presumed fell asleep). Assumed sleep (the sleep period) represents the duration from fell asleep to the end of the last part/block of immobility before getting out of bed (woke up). Fragmentation Index is used to measure sleep quality, composed of the percentage of measured mobile time and immobile bouts with a duration ≤1 min, during assumed sleep. Wake bouts means single episodes of wakefulness during the sleep period. Total daytime activity score was defined by the sum of the activity counts during wakefulness. Actigraphy has been validated as an objective measure of sleep and physical activity in pediatric populations. Validation studies in middle childhood (ages 5–12 years) demonstrate acceptable agreement with polysomnography (PSG) for key sleep parameters, with high sensitivity for detecting sleep but poor specificity for detecting wakefulness (59). More recent research in children and adolescents aged 8–16 years has evaluated multiple scoring algorithms and device placements relative to PSG, providing more nuanced insight into the accuracy and limitations of actigraphic sleep metrics (60). In addition, normative reference values for actigraphic motor activity during sleep in children and adolescents have been established, offering valuable comparative benchmarks (61). Actigraphy has also been evaluated in young children with ASD, demonstrating good reliability for most sleep parameters when compared with PSG (62).

### Physical activity program

Following parent questionnaires, cognitive assessment and actigraphy monitoring, children were invited to join a 10–12 weeks physical activity program. The main purpose of the intervention was pulse-raising (aerobic) activity, performed either at home with parents or in a group setting arranged by the research team. All participants were included in a single intervention group regardless of the type of exercise. Attendance and participation were verified using a training diary at home and a session log maintained by the instructors. The children were asked to take part in training up to three times per week and were encouraged to wear a pulse watch during the intervention period. Children who trained at home were provided with an instructional video featuring a physical training program and relaxation exercise, a yoga mat and gym balls. Group training sessions were held either at a private training facility (Friskis and Svettis, Lund) or in gymnastic halls at the Child and Adolescent Habilitation Clinics in Lund and Malmö. Groups were small, typically consisting of two to three instructors and one to five children per session, with the option for individual training in a separate room with one instructor. Students from the physiotherapy program at Lund University were recruited to serve as personal trainers. Each trainer was assigned one or two children for whom they were specifically responsible for during the intervention. Each training session lasted in total approximately one hour and followed the following format: beginning with a 10 min warm-up, followed by 40 min of exercises, primarily circuit training using bodyweight or ball activities, and ending with a 10 min relaxation to conclude the session. To enhance motivation among the children, the content, intensity, and difficulty of the exercises were varied. The primary goal was to ensure that the activities were both pulse-raising and enjoyable. The children were encouraged to suggest their own exercises, and the program was tailored to accommodate each child’s individual abilities and needs. Team sports and competitive activities were not included. Within three months after completing the intervention, children were invited to repeat the assessment questionnaires, cognitive test and actigraphy.

### Statistics

Statistical analyses were conducted using IBM SPSS Statistics for Windows, version 29.0. Comparative analyses between all initially included participants and the intervention group (in **Table 1**) were done using the chi-square-test. Histogram of pre- and post-intervention test scores (parent questionnaires, CANTAB tests and actigraphy) were generated to assess normal or skewed distribution of data. In borderline cases, the Shapiro Wilk test and Q-Q plots were performed to evaluate normality. Pre- and post-intervention results were compared using a parametric paired sample t-test for normally distributed data and presented as mean, standard deviation (SD), and number of samples (n). P-values ≤0.05 were considered statistically significant. Effect sizes were calculated with Cohen´s D test.

**Table 1:** Demographics and reported medical history.

Variable	All participants	Participants in the intervention group
	n=30	n=16
Demography		
Age: median years (range)	12.0 (9–14)	11.5 (9–13)
Gender: boys % (n)	73.3 (22)	75.0 (12)
Reported medical history		
ADHD or ADD _1_% (n)	80.0 (24)	75.0 (12)
Intellectual disability _2_% (n)	3.3 (1)	0 (0)
Other psychiatric diagnosis _3_% (n)	40.0 (12)	18.7 (3)
Prematurity _4_% (n) -Missing data	13.3 (4) 16.7 (5)	23.1 (3) 7.7 (1)
Epilepsy _5_% (n)	23.3 (7)	18.7 (3)
Other somatic diagnosis _6_% (n)	26.7 (8)	12.5 (2)
MRT of brain % (n) -Findings _7_ -No findings -Missing data	10.0 (3) 10.0 (3) 80.0 (24)	0 (0) 12.5 (2) 87.5 (14)
Schooling % (n) -Regular school with no support -Regular school with support -Adapted school form -Missing data	16.7 (5) 46.7 (14) 20.0 (6) 16.7 (5)	18.7 (3) 37.5 (6) 25.0 (4) 18.7 (3)

Demographics and reported medical history of participants with high functioning ASD. _1_ n = 3 ADD, n = 21 ADHD. _2_ n=1 mild intellectual disability. _3_ n=2 major depressive disorder, n=1 major depressive disorder and eating disorder, n=2 anxiety disorder, n=1 obsessive compulsive disorder, n=1 anxiety disorder, obsessive compulsive disorder and tics, n=1 anxiety disorder and developmental coordination disorder, n=1 non-organic sleeping disorder, n=1 pragmatic language disorder, n=1 non-organic enuresis, n=1 impressive and expressive language disorder, specific reading disorder and non-organic enuresis. _4_ n=1 born in gestational week 27 + 5 with intraventricular cerebral hemorrage and posthemorragic hydrocephalus, n=1 twin pregnancy, born gestational week 35 + 6, n=2 twin pregnancy, born gestational week 36 + 4. _5_ n=4 focal epilepsy, n=3 generalized epilepsy. _6_ n=2 migraine, n=1 celiac disease, n=1 psoriasis, n=1 hydrocephalus, n=1 lumbosacral dermal sinus, psoriasis and neuromuscular bladder disorder, n=1 unilateral hearing loss, n=1 bilateral sensorineural hearing loss, n=1 allergic rhinitis. _7_ n=1 enlarged ventricles, reduction in white substance and subcortical volume reduction, n=1 grey substance heterotopia, n=1 cortical dysplasia. Missing data indicate that the information could not be found in the participant´s patient record, which was the source of data collection.

## Results

### Patient inclusion and clinical characteristics

In total, 30 children diagnosed with ASD level 1-2 (n=28 level 1 and n=2 level 2) were included in the study (**Table 1**). All children lived at home with one or two parents. Their median age was 12 years and the majority were boys (n=22; 73.3%). Comorbidities included primarily ADHD, but also other psychiatric disorders, epilepsy and other somatic disorders. Most of the children had never done a MRT of the brain, probably due to lack of clinical indication. Due to their underlying neuropsychiatric condition, the children typically received additional support or adaptations in school. **Figure 1** provides a flowchart summarizing participant engagement and dropout rates at each study stage. Out of n=30 included children, n=16 completed the physical activity intervention program, including n=2 with ASD level 2. N=11 children opted out prior to the physical training program, mainly due to practical issues. Additional n=3 withdrew shortly after beginning, citing primarily high stress levels/unwillingness related to underlying challenges/comorbidities. The intervention group (n = 16) is referred to as children with high-functioning autism, as all participants had ASD Level 1–2, spoken language, and no intellectual disability. Out of n=12 initial children with psychiatric comorbidity, n=3 chose to participate in the intervention, and of the n=15 initial children with epilepsy or other somatic comorbidity, n=5 took part in intervention. No statistically significant differences were observed in the prevalence of comorbidities, including ADHD/Attention deficit disorder (ADD) (p = 0.69), other psychiatric disorders (p = 0.14), epilepsy (p = 0.72), or other somatic disorders (p = 0.27), between the initially included children (n=30) and those who continued with the physical training program (n=16) (**Table 1**).

Parents were asked to complete a baseline questionnaire regarding their child’s physical activity levels, sleep habits, and ongoing pharmacological treatments when performing the actigraphy pre-intervention. Henceforth, the characteristics of the intervention group (n=16) who completed the physical training program is described. Estimated level of physical activity pre-intervention was low in most of the children (n=9 out of 16 never engaged in any physical training, n=2 participated in physical training once-twice/week, n=2 3–5 times/week, n=1 ≥5 times/week, n=2 missing data). Estimated time spent with low-intensity physical activity per week pre-intervention ranged from 50 to >150 min/week (n=7 50–99 min/week, n=3 100–150 min/week, n=4 >150 min/week, n=2 missing data). N = 4 out of 16 children had no prior experience with organized physical training activities, and only n=3 were involved in physical training outside of school at the beginning of the study. Leisure activities were reported to be largely sedentary for n=9.

Regarding sleep habits only n=3 out of 16 children in the intervention group reported sleep disturbances (n=1 missing data), although the majority were taking sleep medications (n=10 melatonin). In addition, n=4 had guanfacine, n=8 central-stimulating drugs, n=2 risperidone, n=3 antiepileptic medications (lamotrigine, valproic acid and levetiracetam), and n=3 antidepressant medications (sertraline and atomoxetine). N = 3 had no medication.

For *post hoc* sub-group analyses, the intervention group was evenly divided into a high-attendance group (n=8; all boys) with physical training program ≥2–3 times per week, and a low-attendance group (n=8; n=4 girls and n=4 boys) with 1-<2 times per week. The attendance data was based on parent reports from training at home, attendance list kept by the trainers in the group trainings, training diary, and pulse watch registrations during training sessions.

### Improved parent-reported executive function in children with autism after 10–12 weeks of physical training

Results from parent questionnaires in the intervention group are presented in **Table 2**. For further description of the questionnaires, see **Supplementary Table 2**. Each domain includes multiple items. Mean ASSQ score did not differ between pre- and post-training assessments (19.8 vs 20.8). Scores above 19 indicate ASD-related clinical difficulties, suggesting that the children exhibited parent-rated ASD symptoms, though as a group just above the clinical threshold.

**Table 2:** Parent-reported questionnaires before and after physical activity intervention program.

Questionnaire	Domaine	N	Mean ± SD pre-intervention	Mean ± SD post-intervention	Two-tailed p-value	Reference value
ASSQ	Total	15	19.8 ± 6.8	20.8 ± 8.6	0.50	>19 indicates clinical level of ASD symptoms
BRIEF-2	Behavioral regulation index	15	67.1 ± 9.7	67.3 ± 11.0	0.92	60–64 mildly elevated; 65–69 potentially clinically elevated; ≥70 clinically elevated difficulties
	Emotional regulation index	14	71.0 ± 10.2	68.6 ± 11.0	0.26	
	Cognitive regulation index	15	70.7 ± 10.2	68.1 ± 7.8	0.12	
	Working memory		71.9 ± 10.7	67.5 ± 9.4	**0.04**	
	Plan/organize		69.4 ± 9.7	65.6 ± 8.3	**0.03**	
	Global executive composite	15	73.5 ± 9.3	70.5 ± 8.2	**0.04**	
CSP	*Quadrants*					Normal interval
	Seeking sensory input	11	45.4 ± 18.0	40.4 ± 17.7	0.08	6–39
	Avoiding sensory input	10	63.2 ± 15.2	57.8 ± 12.5	0.08	9–44
	Sensitivity to sensory input	11	55.4 ± 15.4	50.1 ± 15.8	**0.03**	7–37
	Reduced registration of sensory input	11	55.8 ± 13.2	47.5 ± 16.9	**0.01**	5–40
	*Sensory section*					
	Body position	15	18.5 ± 5.5	14.7 ± 7.8	**0.03**	0-13
	*Behavioral section*					
	Conduct	15	22.4 ± 6.1	19.7 ± 7.4	**0.02**	2-19
SRS	Total	15	78.2 ± 10.2	76.6 ± 8.6	0.31	60–75 mild to moderate social impairment; ≥76 severe social impairment
SDQ						Average difficulties
	Emotional symptoms	14	4.9 ± 2.8	4.4 ± 2.7	0.49	0 - 3
	Conduct problems	13	2.6 ± 1.9	2.5 ± 2.1	0.44	0 - 2
	Hyperactivity/inattention	14	6.2 ± 2.4	6.1 ± 2.8	0.73	0 - 5
	Peer relationship problems	14	5.8 ± 2.1	5.2 ± 2.2	0.23	0 - 2
	Prosocial behaviour	14	7.1 ± 2.5	6.4 ± 2.8	**0.03**	9 - 10
	Problems in everyday life	14	5.1 ± 2.1	4.9 ± 2.9	0.70	0
	Total	13	19.8 ± 5.1	18.6 ± 6.4	0.25	0-13
SNAP-IV	Combined inattention + hyperactivity/impulsivity	15	1.67 ± 0.51	1.62 ± 0.56	0.50	≥ 1.67 indicates clinical difficulties

Parent-reported questionnaires before and after physical activity intervention program for children with high functioning ASD. Within the parent questionnaires, some items were left unanswered, hence, number of participants (n) varies between different items. Statistically significant values (p < 0.05) are presented in bold.

In the BRIEF-2 questionnaire, higher scores indicate increasing levels of difficulty, with scores ≥70 considered to represent clinically elevated difficulties. Pre-intervention ratings indicated ≥70 in 4/6 domains, whereas post-intervention only 1/6 remained at this level. Significant statistical improvement were observed in three domains: the Working Memory (WM) and Planning/Organizing (P/O) within the Cognitive Regulation Index and the Global Executive Composite (GEC), the latter representing an average score across all domains. WM, P/O and GEC showed pre-intervention average scores indicating clinically or potentially clinically elevated difficulties, which dropped by 3–4 steps post-intervention. The changes corresponded to moderate effect sizes (d = 0.58, 0.61, 0.58, respectively), but the analyses were underpowered (statistical power = 0.55, 0.6, 0.55). When the intervention group was subdivided based on training attendance, statistical significance was no longer reached.

In the CSP questionnaire, domain scores are compared to normative data and categorized by standard deviations from the age-matched norm. Thus, improvement in this context are reflected by scores shifting closer to age-normative values. None of the scores, either pre- or post-intervention, fell within the normal range, remaining one or two SD above normal interval. However, two quadrants demonstrated significant improvement post-training: Sensitivity to sensory input and Reduced registration of sensory input (moderate-large effect size d = 0.73 and 0.93, moderate and adequate statistical power = 0.59 and 0.79). Additionally, in the Sensory section, Body position related to sensory processing improved and in the Behavioral section, Conduct related to sensory processing improved as well (moderate effect sizes d = 0.61 and 0.66, statistical power = 0.60 and 0.66). When subgrouping for training attendance, significant changes remained for the low-attendance group, regarding Sensitivity to sensory input (n=6, mean pre-intervention = 59.2 ± 17.4 vs post-intervention = 50.5 ± 19.1, p = 0.01), Reduced registration of sensory input (n=6, mean pre-intervention = 54.0 ± 16.1 vs post-intervention = 41.8 ± 19.4, p = 0.03), and Body position related to sensory processing (n=8, mean pre-intervention = 19.4 ± 4.3 vs post-intervention = 11.9 ± 8.1, p = 0.01).

No differences were found in the SRS total score or in any of its subscales. The total average score was within reference values for severe social impairments both before and after the training period. Similarly, the SDQ results showed average rating of symptoms and problems above or outside the age norm across all domains, with no differences pre- and post-intervention. An exception was prosocial behavior where the group started below the age average before training and showed a further significant decline after the training period (moderate effect size d = 0.63, statistical power = 0.58). This decline remained significant in the subgroup low-attendance group (n=7, mean pre-intervention = 7.9 ± 2.3 vs post-intervention = 7.3 ± 2.4, p = 0.03). Finally, no differences were found in SNAP-IV scores for combined inattention and hyperactivity/impulsivity, including when subgrouping for co-occurring ADHD (n=11, mean pre-intervention = 1.8 ± 0.5 vs post-intervention = 1.8 ± 0.5, p= 0.79).

### Improved spatial planning and problem-solving strategy in children with autism after 10–12 weeks of physical training

Five CANTAB tests were assessed pre- and post-intervention: IED, PAL, RTI, SOC, and SWM (**Table 3**). For description of each test, see **Supplementary Table 3**. Only one test, SOC, showed statistic significant differences for multiple items pre- vs post-intervention. A significant decrease was observed post-training in SOC initial and subsequent thinking time at different levels of difficulties (SOCITMD3, SOCSTMD4 and SOCSTMD5), suggesting an improvement (moderate-large effect sizes d = 0.71, 0.80, 0.80, slightly underpowered analyses with statistical power = 0.65, 0.75, 0.76, respectively). SOCITMD provides an indication of the time taken to plan problem solution, discounting movement time, and SOCSTMD the time taken by the subject to plan or re-plan problem solution. When subgrouping for training attendance, significant improvement was still present in the low-attendance group for SOCITMD3 (n=7, mean pre-intervention = 3.5 ± 1.7 vs post-intervention = 2.0 ± 0.9, p=0.04).

**Table 3:** Cognitive assessment with CANTAB before and after physical activity intervention program.

Test	Outcome measurement	Description	n	Mean ± SD pre-intervention	Mean ± SD post-intervention	Min-max	Two-tailed p
IED	IEDYCOST	Stages completed (no)	12	8.1 ± 1.7	8.2 ± 1.4	0–9	0.67
	IEDYEARTA	Total errors, adjusted (no)	13	46.8 ± 46.8	38.5 ± 39.0	0–402	0.30
	IEDTTA	Total trials, adjusted (no)	13	136.1 ± 83.7	122.6 ± 67.9	54–450	0.33
PAL	PALNPR212	Number of patterns reached (no)	14	11.7 ± 1.1	12.0 ± 0.0	2–12	0.34
	PALTEA28	Total errors, adjusted (no)	14	7.9 ± 8.3	5.2 ± 3.5	0–70	0.25
	PALMETS28	Mean errors to success (no)	14	1.6 ± 1.2	1.2 ± 1.0	0–15	0.21
RTI	RTIFESIL	Five-choice error; incorrect (no)	15	0.0 ± 0.0	0.1 ± 0.3	0–30	0.33
	RTIFESNR	Five- choice error; no response (no)	15	0.4 ± 1.1	0.2 ± 0.4	0–30	0.51
	RTIFESPR	Five-choice error; premature (no)	15	0.9 ± 1.7	0.7 ± 1.4	0–30	0.76
SOC	SOCPSMMT	Problems solved in minimum moves; total (no)	13	7.8 ± 1.8	8.6 ± 2.1	0–12	0.28
	SOCITMD2	Initial thinking time, median of 2–5 moves, sec	13	2.1 ± 2.8	1.5 ± 1.1	0–	0.46
	SOCITMD3		13	3.6 ± 1.6	2.2 ± 1.4	0–	**0.02**
	SOCITMD4		13	3.0 ± 1.6	2.7 ± 1.8	0–	0.62
	SOCITMD5		13	3.9 ± 3.8	3.3 ± 4.5	0–	0.68
	SOCSTMD2	Subsequent thinking time, median of 2–5 moves, msec	13	321 ± 642	305 ± 458	0–	0.95
	SOCSTMD3		13	424 ± 606	163 ± 341	0–	0.17
	SOCSTMD4		13	285 ± 404	84 ± 242	0–	**0.01**
	SOCSTMD5		13	532 ± 641	57 ± 97	0–	**0.01**
SWM	SWMPR	Problem reached (no)	14	7.0 ± 0.0	7.0 ± 0.0	3–7	-
	SWMTE468	Total errors (no)	14	10.1 ± 7.0	13.2 ± 9.7	0–157	0.22
	SWMS	Strategy in search pattern for 6–8 boxes (no)	14	7.9 ± 2.0	7.7 ± 2.3	2–14	0.84

Cognitive assessment with CANTAB before and after physical activity intervention program for children with high functioning ASD. During assessment some children did not fully complete the tasks due to lack of energy or motivation, hence, number of participants (n) varies between different items. Statistically significant values (p < 0.05) are presented in bold.

### More cohesive sleep with fewer awakenings in children with autism after 10–12 weeks of physical training

**Figure 2:**
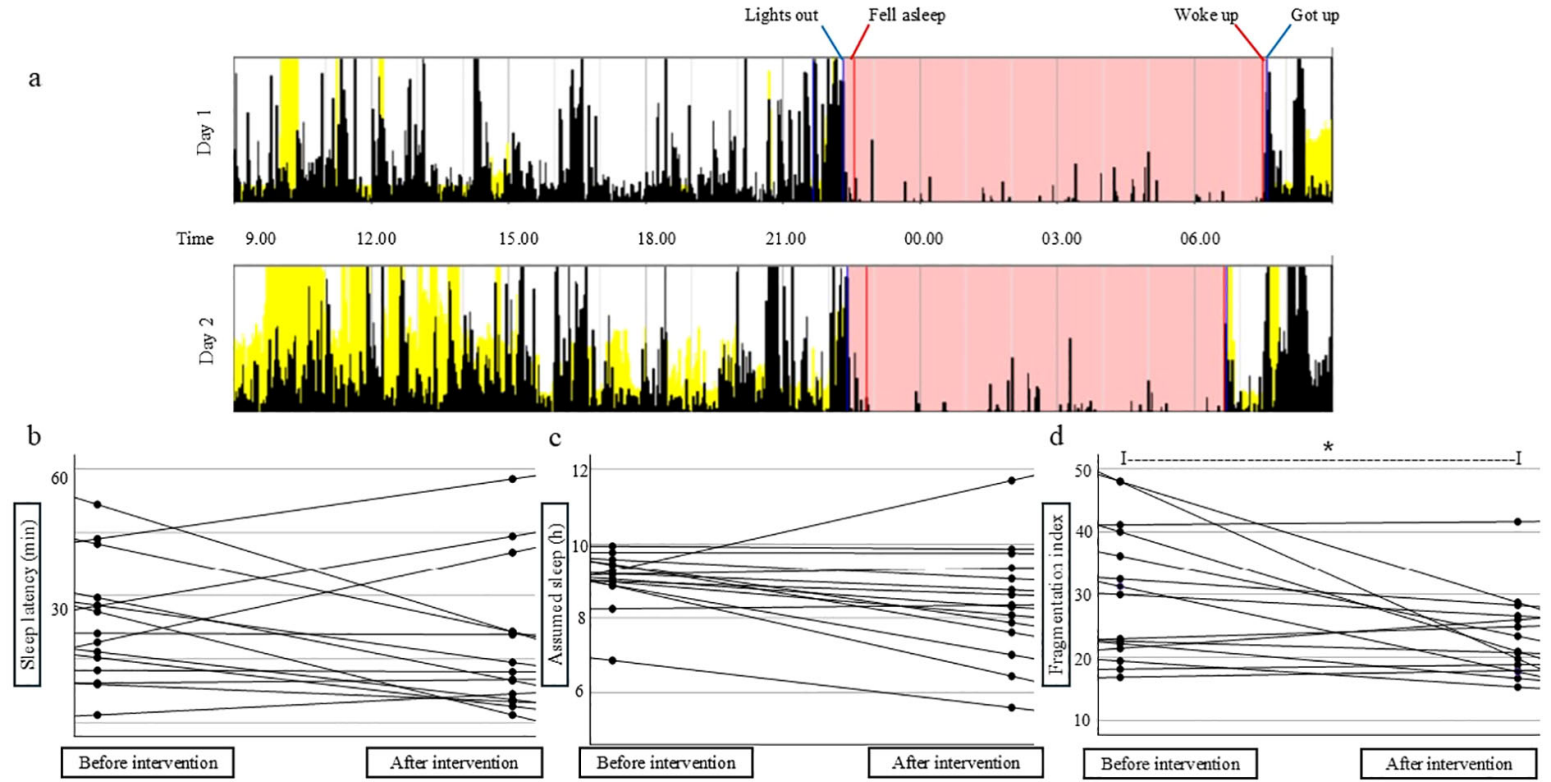
Actigraphy monitoring of sleep patterns and daytime activity before and after physical activity intervention program for children with high functioning ASD. **(a)** Example of actigram in the Motionware software, showing sleep- and wake periods over 2 days. White: wake period. Pink: time in bed. Yellow: light registration. The Lights out event (blue marker) can also be seen as a cessation of daylight (yellow). Fell asleep (red marker) is when movement ceases during time in bed. Woke up (red marker): start of last sustained movement before the Got up event (blue marker) at the beginning of the wake period. In **(a)** the Woke up and Got up events coincide, most likely due to a set alarm clock. **(b)** Sleep latency (from Lights out to Fell asleep). **(c)** Assumed sleep (from Fell asleep to Woke up) **(d)** Fragmentation index (percentage of mobile and immobile bouts during assumed sleep) before and after the physical training program. The actigraphy measurement were based on 72 hrs of recordings during 3 weekdays pre/post-intervention in n=16 children, with the following limitations: pre-intervention n=3 had two days and n-3 one day of daytime recording. Post-intervention n=4 had no daytime recordings, n=1 two daytime recordings, and n=6 had 3-15.5 hrs of daytime recordings in total during 3 days. Regarding nighttime recordings, pre-intervention n=3 children had two nights and n=1 had one night of recordings. Post-intervention, n=1 child had one night of recordings. Reasons for removal of the actigraphy device included i.e. bathing, perceived discomfort, or misunderstanding of instructions to wear the device around the clock. N=2 children performed the actigraphy 4 months post-intervention, slightly beyond the planned 3-month period, due to practical challenges and stress-related factors. Data presented as individual dots for each participant in min **(b)**, hours **(c)** and index units **(d)**. *p=0.01 in d.

**Figure 2a** shows an example of actigram in the Motionware software, showing sleep- and wake periods over 2 days. The median bedtime (median Lights out) reported by participants was 9:10 pm (range 8:13 -10:17 pm) before and 9:47 pm (range 7 - 10:49 pm + single cases 11:38 pm, 00:31 am, and 04:06 am) after attending the training program. Sleep latency, the time from going to bed until presumed falling asleep, did not change for the intervention group post-training (n=15, **Figure 2b**). However, among the subgroup of children who reported taking melatonin before bed during the actigraphy assessment, sleep latency was significantly decreased after the training period (n=7, mean pre-intervention = 21 ± 8.1 min vs post-intervention = 10 ± 7.0 min, p = 0.05).

The average assumed sleep duration showed no significant difference after the training period (**Figure 2c**). There were also no differences in the duration of single episodes of waking/wake bouts (n=15, mean pre-intervention = 171.7 ± 41.6 sec vs post-intervention = 201.2 ± 221.8 sec, p = 0.62). However, the fragmentation index, a measure of movements or possible waking/arousals during the entire assumed sleep period, significantly decreased in the intervention group after the training period (n=15, mean pre-intervention = 30.0 ± 10.6 vs mean post-intervention = 23.1 ± 6.7, moderate-large effect size d = 0.72, slightly underpowered analyses with statistical power = 0.74) (**Figure 2d**). This reduction did not persist when participants were further subdivided into low- and high-training attendance (data not shown).

No change in the total activity score while awake during daytime was observed in the intervention group after completing the training program (n=13, mean pre-intervention = 284625 ± 94255 vs post-intervention = 271322 ± 88748 score, p = 0.57).

## Discussion

This study provides evidence for a beneficial effect of physical activity on cognition and sleep in children aged 10–14 years diagnosed with high-functioning ASD. Parent-rated questionnaires assessing psychological symptoms indicated significant improvements in executive function, particularly in working memory and planning/organizing abilities, following 10–12 weeks of physical training. The questionnaires also suggested reductions in difficulties related to sensory sensitivity and sensory input registration. Objective assessments using the CANTAB cognitive battery tentatively suggested improvements in executive function. Specifically, the SOC test, which measures planning and problem-solving strategies, showed gains in initial and subsequent thinking time at certain difficulty levels, although these findings should be interpreted with caution due to the small sample size and the fact that improvements were observed only in some of the measures. Actigraphy recordings of locomotor activity during presumed sleep and awake revealed a decrease in the sleep fragmentation index after participation in the physical activity program, indicating fewer nocturnal awakenings and more consolidated sleep. In addition, a reduction in sleep latency was observed in a subgroup of children with regular melatonin intake.

The observed enhancement in parent-reported executive function, particularly in WM and P/O, showed a moderate effect size, although the study was statistically underpowered. This finding is consistent with a meta-analysis of 24 studies (n = 944) examining the effects of exercise on executive function in healthy individuals aged 6–35 years (19). In that analysis, the largest effect size was reported for inhibition, followed by planning and working memory. However, the authors cautioned that the results for working memory and planning should be interpreted carefully due to the limited number of studies available at the time.

Studies investigating the effects of exercise in children with ASD likewise suggest positive impacts on cognition (63–68). Chronic exercise interventions have been associated with small-to-moderate improvements in executive functions in children and adolescents with ASD, particularly in cognitive flexibility and inhibitory control (65, 67). For example, a 12-week table tennis training program, consisting of 70-minute sessions twice per week, improved mental flexibility as measured by the computerized Wisconsin Card Sorting Test (WCST) in children aged 6–12 years with high-functioning ASD (66). Cognitive engagement has also been identified as a key factor, as shown by Tse et al. (68), where children with autism who learned to ride a bicycle demonstrated improvements across four executive function components (planning, working memory, flexibility, and inhibition), while no improvements were observed in a stationary bicycle or control group. In the present study, training sessions were individualized according to participants’ needs and preferences, which may have enhanced both study adherence and cognitive outcomes. A common limitation across these studies, including ours, is the relatively small sample size, underscoring the challenges inherent in conducting intervention research in this population.

The moderate-to-large effect sizes for improvements in spatial planning and problem-solving strategies observed in the present study, as measured objectively using parts of the CANTAB battery, support the findings from parent-rated questionnaires and further suggest that physical activity programs may serve as beneficial strategies for enhancing executive functions in children with ASD. The CANTAB has previously been validated for use in children with ASD (54). It has also been employed to detect improvements in spatial planning and working memory in healthy children following an intensive physical exercise program (69), and in a more recent study involving a large cohort of 426 healthy children aged 7–12 years, in which working memory improved following various physical activity interventions, including team sports, aerobic exercise, and coordinative exercise (70). Combining subjective and objective evaluations of executive functions offers an effective approach for corroborating individual findings and reducing potential subjective bias.

The majority of children in the present study had comorbid ADHD/ADD, and executive difficulties are associated with both diagnoses. However, no improvements were observed in the SNAP-IV questionnaire, which assesses ADHD-related symptoms such as inattention and hyperactivity/impulsivity. This contrasts with findings from a recent study of children with ADHD, in which a 20-session exercise intervention resulted in improvements on both the BRIEF and SNAP-IV questionnaires (71). In a meta-analytic review of six studies including individuals with ASD and/or ADHD aged 3–25 years, Tan et al. (67) evaluated the efficacy of physical exercise interventions on cognitive functions and reported overall small-to-moderate effects across certain domains, comparable to effect sizes observed in healthy individuals (19). Similarly, another meta-analysis of 11 studies involving participants with ADHD and/or ASD concluded that physical activity interventions can promote motor skills and executive functions, particularly inhibitory control, cognitive flexibility, and gross motor skills (72). In summary, although the current research field is fragmented, available evidence suggests that children with ASD and comorbid ADHD may benefit from physical exercise with respect to cognitive function, even in the absence of significant improvements in attention or hyperactivity/impulsivity.

Parents in the present study reported improvements on several CSP items related to sensory processing following the physical activity program, with moderate-to-large effect sizes and adequate statistical power. These findings are partly consistent with those of Bass et al. (73), who reported improvements in sensory integration and sensitivity, assessed by the CSP, following horseback riding in children with ASD. However, in contrast to Bass et al. (73), parents in the present study did not report improvements in social impairment, such as social motivation, as assessed by the SRS questionnaire after the intervention. Notably, the current cohort, which scored below age-average levels on prosocial behavior according to the SDQ questionnaire, showed a slight further decline following the training period. Although this isolated finding demonstrated a moderate effect size, the analysis was statistically underpowered. Nevertheless, the result is noteworthy and may be attributable to increased energy demands associated with engagement in the physical activity program, potentially resulting in fewer social interactions outside of training.

Cognitive function may be adversely affected by poor sleep in children with ASD (74). Physical activity interventions have shown promising effects on sleep quality in this population. A recent meta-analysis of eight studies reported large beneficial effects of exercise on parent-reported general sleep problems in children and adolescents with ASD (75). In the present study, however, subjective sleep parameters were difficult to evaluate, as only three children were reported to have sleep problems, although the majority were receiving sleep medications.

With respect to actigraphy-measured parameters, two previous studies demonstrated improved sleep quality in children with ASD following 10 weeks of morning jogging (76) and after a 12-week basketball skills program (33). These studies, which included both sleep logs and actigraphy, reported improvements in sleep efficiency, sleep onset latency, sleep duration, and wake after sleep onset. In contrast, multiple improvements in sleep parameters were not evident in the present study. Nevertheless, a reduction in sleep fragmentation following the intervention showed a moderate-to-large effect size with borderline adequate statistical power, suggesting more consolidated sleep.

Assessment of sleep onset latency was confounded by the use of sleep medications. Still, a significant reduction was observed in children who regularly took melatonin, with latency reduced by approximately 50%. The result may indicate that the children who struggled the most with falling asleep gained the most from the intervention. Seasonal variations in daylight may have represented another confounding factor, as the intervention was initiated during autumn when daylight hours progressively decrease.

The assumed sleep duration was within the normal range, and total daytime activity scores did not change following the intervention, indicating no major alterations in circadian rhythm across the recorded representative weekdays. Previous research has shown that actigraphy may register greater motor activity during sleep in children with ASD, including those with comorbid ADHD, compared to typically developing children (77). Future larger cohort studies with longer actigraphy recordings are warranted to clarify the potential effects of physical activity on this presumed sleep restlessness and on the regulation of sleep–wake cycles.

The amount of physical activity may be associated with greater improvements in both sleep quality and performance (78), and high-intensity exercise programs for children with ASD, when attended regularly, have been correlated with better physical performance and higher satisfaction (79). In the present cohort, subdivision into low- and high-attendance groups confirmed isolated statistical changes in executive and sensory functions in the low-attendance group, although interpretation is limited by the small sample size. However, the relative change in physical activity levels from pre- to post-intervention was not calculated, meaning that a child in the low-attendance group could nevertheless have experienced a greater relative increase in activity than a child in the high-attendance group. More standardized pre-intervention assessments of baseline physical activity levels, in addition to estimates of activity during the intervention, are recommended for future studies to enable more accurate comparisons of outcomes across different types and intensities of physical activity programs.

In the present study, families were allowed to choose between on-site group training and home-based training in order to facilitate participation and enhance adherence. Due to small subgroup sizes and variability in training format (on-site only, home-based only, or a combination of both), no comparisons between training formats were performed. To our knowledge, no previous studies have directly compared on-site versus home-based physical training interventions in children with ASD. However, in a study of children with obesity, Lisón et al. (80) compared a hospital-based group exercise program (n = 45) with a home-based program (n = 41) and found comparable attendance rates between groups, suggesting that home-based exercise may represent a feasible and effective alternative to on-site group training for children and adolescents. Nevertheless, further research is needed to evaluate and compare different training formats, both in pediatric populations in general and specifically in children with ASD.

### Strengths and limitations

A strength of the present study is that parent-rated ASSQ scores were only slightly above the recommended cutoff for ASD, indicating a low level of parent-reported autistic traits in the cohort. This supports that the intervention group consisted predominantly of children with high-functioning ASD, that is, ASD without intellectual disability and with lower support needs (corresponding to DSM-5 levels 1–2). Based on estimated pre-intervention physical activity levels, the cohort was predominantly sedentary at baseline, rendering it representative of the broader population of children with autism. Most participants did not engage in sports at school or during their leisure time. Although the study experienced a considerable dropout rate prior to the start of the intervention, the majority of children who initiated the physical activity program completed it. This suggests that the adaptation of training sessions to the individual needs of participants was successful.

Another strength of the present study is the representative comorbidity profile, particularly the high prevalence of ADHD. Approximately 75–80% of participants had a diagnosis of ADHD, consistent with previously reported co-occurrence rates of ADHD symptoms in individuals with ASD, which range from 30% to 85% depending on the clinical sample (6, 81–85). The gender ratio was also consistent with recent research suggesting a male-to-female ratio closer to 3:1 for ASD, rather than the traditionally assumed 4:1 (86).

Finally, an additional strength of the study lies in the inclusion of both parent-reported measures and cognitive assessments to evaluate the intervention, with both indicating improvements in executive functions.

A limitation of the study is the potential for selection bias, which was unavoidable given the study design. Participation required parents to accompany their children to multiple assessments and training sessions, which posed a barrier for many families who were already overburdened. To improve compliance, the research team provided detailed descriptions of each study step and employed a low-arousal approach in interactions with participants and families.

Another limitation of the study is the lack of standardized self-reported measures from the children. However, participation already imposed a substantial burden, and adding further tasks could have negatively affected compliance.

A further limitation concerns the actigraphy data, which were collected for only three nights, or fewer for some participants, due to compliance issues. Although three nights have been used in previous studies and considered acceptable, a more precise assessment of day-to-day variation and daytime activity would have required a longer monitoring period, likely at least 5–7 nights (58).

Regarding standardization of the physical activity program, the study encountered difficulties in quantifying and verifying the amount and intensity of training, due to practical issues such as problems with pulse watches and limited compliance in maintaining training diaries. The primary focus was on enrolling participants and encouraging sustained physical activity throughout the 10–12-week program, which partly explains the variation in training frequency, ranging from one to three sessions per week.

Finally, although statistically significant improvements were observed in this exploratory study, most effect sizes were moderate to large, while the analyses were statistically underpowered. Therefore, the findings should be interpreted with caution.

## Conclusions

In this exploratory study, preadolescents with high-functioning ASD (primarily level 1 and without intellectual disability) demonstrated improvements in cognitive functions, including executive functions and sensory processing, as well as more coherent sleep patterns following participation in a structured 10–12-week physical activity program. Further randomized investigations are now warranted, given that children with ASD are often more sedentary than their peers. This inactivity may persist into adulthood, where individuals with ASD have been reported to perceive fewer benefits of exercise and to offer more rationalizations for avoiding physical activity (87). Therefore, early implementation of targeted physical activity interventions, such as the one examined in the present study, may yield significant developmental benefits for children with ASD.

## Data Availability

The raw data supporting the conclusions of this article will be made available by the authors, without undue reservation.
