# Supplementary material for: Improved executive function and sleep quality in preteens with high-functioning autism following a structured physical activity program
Source: Front Psychiatry. 2026 Mar 16;16:1726809. doi: 10.3389/fpsyt.2025.1726809 (PMC13033741; doi:10.3389/fpsyt.2025.1726809)
Supplement: Supplementary file 1 [file Table1.docx]

**Supplementary Table 1.** Description of Parent Questionnaires

| **ASSQ** is a screening questionnaire for autism spectrum disorder (ASD), containing 27 questions or statements assessing behaviors related to ASD. The ratings available are “no” (0 point), “somewhat” (1 point), “yes” (2 points). The points are added up to a total score of maximum 54 points. For a clinical population the score 19 are used as a cutoff to indicate ASD. The psychometric properties have been studied in several publications showing a good test-retest reliability for parent ratings (0.96) and a sensitivity of 0.91 and specificity of 0.86 in a Norwegian study on school children aged 7-9, using 17 points as cutoff (Ehlers et al., 1999; Ehlers & Gillberg, 1993; Kopp & Gillberg, 2011; Posserud et al., 2006). |
| --- |
| **BRIEF** is a questionnaire measuring executive functions and self-regulation in children and adolescents ages 5-18 in the parent form. The questionnaire consists of 63 statements and nine clinical scales: inhibition, self-monitor, shift, emotional control, initiate, working memory, plan/organize, task-monitor and organizing of materials. These scales are distributed under three domains: behavioral index (inhibition, self-monitor), emotional index (shift, emotional control) and cognitive index (initiate, working memory, plan/organize, task-monitor, organizing of materials). The global executive composite (GEC) averages all assessment values. The rater records their answer via a Likert-type format N (never), S (sometimes) or O (often). Results are presented in T-score format (M=50, SD=10) and a higher T-score suggests that the child exhibits significant difficulties with executive functions. The results could also be compared to age-matched norms. The psychometric properties have been evaluated in children and youths with and without ADHD and ASD (Gioia et al., 2002; LeJeune et al., 2010). |
| **CSP** is an assessment for evaluating a child’s sensory processing patterns in different environments and how they impact daily functioning. The parent form can be used for children ages 3-14 years. It consists of 86 statements and the parents record their answer by choosing between “almost always”, “frequently”, “half of the time”, “occasionally”, “almost never” or “does not apply”. Most of the items are counted under one of the quadrants: sensation seeking (child seeks sensory input), sensation avoiding (child is bothered by sensory input), sensory sensitivity (child is sensitive to sensory input) and low registration (child misses sensory input). The form also provides information about how the child processes different modalities of sensory inputs divided into the following sensory sections: auditory, visual, touch, movement, body position and oral. Behavioral responses related to sensory inputs are divided into the following behavioral sections: conduct associated with sensory processing, social emotional responses associated with sensory processing and attentional responses associated with sensory processing. The results are compared to a normal distribution scale and categorized in standard deviations from the norm. Psychometric properties have been evaluated in typically developed children and children with ASD (Dunn, 1994; Dunn & Westman, 1997; Dunn & Brown, 1997; Tomchek & Dunn, 2007). |
| **SRS** aims to identify social impairment associated with ASD and quantify its severity. The school age form applies for parents to children and adolescents ages 4-18 years. It consists of 65 items which are rated on a graduated response scale: “never true”, “sometimes true”, “often true” and “almost always true”. Five subscales are used for evaluating treatment: social awareness, social cognition, social communication, social motivation and restricted interest and repetitive behavior. The subscales are preferably not used for screening or diagnosis. The total and subscale scores are compared to clinical cut-offs indicating presence of autism-related social impairments. Psychometric properties have been evaluated in typically developed children and children with ASD (Constantino et al., 2003). |
| **SDQ** is an emotional and behavioral screening questionnaire for parents to children and adolescents ages 4-17 years. It consists of 33 questions or statements and the responder records their answer for the first 25 items on the response scale: “not true”, “somewhat true” or “certainly true”. The remaining items address the severity and the duration of the difficulties. The first 25 items asses five scales: emotional symptoms, conduct problems, hyperactivity/inattention, peer relationship problems and prosocial behavior. The total score helps to categorize the child or adolescent as having “normal”, “borderline” or “abnormal” difficulties. For the subscales there are clinical cut-off scores. Psychometric properties have been evaluated in larger community samples of children aged 3-17 years (Goodman,1997; Stone et al., 2010). |
| **SNAP-IV** is a 30-item rating scale for parents to assess symptoms of ADHD (inattention and hyperactivity/impulsivity) and symptoms of oppositional defiant disorder, based on the DSM criteria. It is divided into subscales for inattention, hyperactivity/impulsivity, and oppositional behavior. Symptom severity is rated on a 4-point scale: not at all (0), just a little (1), quite a bit (2) and very much (3). Subscale scores are calculated and compared to clinical cut-off scores. Psychometric properties have been evaluated in children with and without ADHD (Bussing et al., 2008; Swanson et al., 2001). |
