# Supplementary material for: Improved executive function and sleep quality in preteens with high-functioning autism following a structured physical activity program
Source: Front Psychiatry. 2026 Mar 16;16:1726809. doi: 10.3389/fpsyt.2025.1726809 (PMC13033741; doi:10.3389/fpsyt.2025.1726809)
Supplement: Supplementary file 2 [file Table2.docx]

**Supplementary Table 2.** Description of selected CANTAB tests and outcome measurements (CANTAB Connect Research: Admin Application User Guide, Cambridge Cognition limited 2022, V1.25)

| **Intra-Extra Dimensional Set Shift (IED)**  This test includes 27 outcome measures to evaluate rule acquisition and reversal, requiring visual discrimination, attentional set formation, maintenance, shifting, and attention flexibility. The participant must work out a rule that determines which stimulus is correct. After six correct responses, the stimuli and/or rule changes. Outcome measure assess the number of stages completed, the numbers of trials completed, the number of errors made and response latency. |
| --- |
| IEDYCOST: Total number of stages (out of nine) the subject completed successfully. |
| IEDYEARTA: Total number of times that the subject chose a wrong stimulus – i.e. one incompatible with the current rule, with adjustment for every stage that was not reached. This is a measure of the subject’s efficiency in attempting the test. Subjects failing at any stage of the test will have had less opportunity to make errors. The adjustment is carried out to compensate for this missing data and provides a more comparable error score to subjects completing all stages of the test. |
| IEDTTA: Number of trials completed on all attempted stages with an adjustment for any stages not reached. |
| **Paired Association Learning (PAL)**  With 21 outcome measures, this test evaluates visual memory and new learning abilities. Boxes are displayed and one or more of them contains a pattern. The patterns are then displayed in the middle and the participant must select the box containing the same pattern. Outcome measurements are stages completed, memory scores, the errors made, and the number of trials required to locate the patterns. |
| PALNPR212: Number of patterns presented to the subject on the last problem they reached. |
| PALTEA28: Number of times the subject chose the incorrect box for a stimulus on assessment problems, plus an adjustment for the estimated number of errors they would have made on any problems, attempts and recalls they did not reach. This measure allows comparisons between performance on errors made across all subjects regardless of those who terminated early and those completing the final stage of the task. In this task variant PALTEA does not include 12 box level to provide a direct comparison to Recommended Standard. |
| PALMETS28: Mean number of attempts made by a subject needed for successful completion of a stage. Does not include 12 box level to provide a direct comparison to Recommended Standard. |
| **Reaction Time (RTI)**  Comprising 21 outcome measures, this test assesses motor and mental response speed, as well as movement time, reaction time, response accuracy, and impulsivity. The participant must select and hold a button while circles are presented on the screen. When a circle with a yellow dot in it appears, the participant must react as fast as possible and select the circle with the dot. Outcome measures are reaction time and movement time. |
| RTIFESIL: Total number of trials where the subject selected an incorrect response stimulus versus the stimulus which actually flashed yellow on screen. Calculated across all assessment trials in which the stimulus could appear in any one of five locations. |
| RTIFESNR: Total number of trials where the subject made no response after the presentation of the target stimulus. Calculated across all assessment trials in which the stimulus could appear in any one of five locations. |
| RTIFESPR  Total number of trials where the subject made a response before the presentation of the target stimulus. Calculated across all assessment trials in which the stimulus could appear in any one of five locations. |
| **Stockings of Cambridge (SOC)**  Featuring 27 outcome measures, this test assesses spatial planning and problem-solving strategy formulation. The participant is requested to move balls to copy a pattern and use as few moves as possible. Movement time is discounted in a part of the task where the participants copy the movements made by the computer which mimics the movements the participant made when solving the original problem. Outcome measures include difficulty level reached, mean moves used and thinking time. |
| SOCPSMMT: Number of assessed problems that the subject successfully completed in the minimum possible number of moves. Calculated over all assessed trials. |
| SOCITMD (2–5): Median difference in the time taken to select the first ball for the same problem in the solve condition compared to the follow condition, calculated across all assessed problems with 2-5 moves. Subjects are encouraged to plan their moves before starting to solve the problems. Therefore, this measure provides an indication of the time taken to plan the problem's solution, discounting movement time. For any given problem, the initial thinking time score may be 0 if the subject is slower in the follow condition. |
| SOCSTMD (2–5): Median difference in time between selecting the first ball and completing the problem in the solve condition minus the corresponding time in the follow condition, divided by the number of moves made in the solve phase. Calculated across all assessed problems with 2-5 moves. This measure provides an indication of any time taken by the subject to plan or re-plan the problem solution after they have made their first move taking into account their movement time, and the number of moves made. For any given problem, the subsequent thinking time score may be 0 if the subject is slower in the follow condition. |
| **Spatial Working Memory**  With 24 outcome measures, this test assesses the retention and manipulation of visuospatial information, providing measures of used strategy and working memory. The participant is requested to select the boxes and using a process of elimination to find a token in each of a number of boxes. The number of boxes gradually increases, and the color and position of the boxes changes between trials. Outcome measures include different type of errors and strategy. |
| SWMPR: Number of problems that the subject reached but did not necessarily complete. |
| SWMTE468: Number of times a box is selected that is certain not to contain a token and therefore should not have been visited by the subject, i.e. between errors + within errors - double errors. Calculated across all assessed four, six and eight token trials. |
| SWMS: Number of times a subject initiates a new search pattern from the same box as before. If the subject consistently initiates searches from the same starting point, it can be inferred that they are employing a deliberate and systematic strategy for locating the tokens. Accordingly, a low score reflects a consistent strategy use (1 = the subjects always begin their search from the same box), whereas a high score indicates a lack of strategic consistency, with searches initiated form various starting boxes. Calculated across assessed trials with 6 tokens or more. |
